# *Canis lupus familiaris* and diclofenac: understanding the potential risks of this association

**DOI:** 10.3389/fvets.2024.1507390

**Published:** 2024-12-09

**Authors:** Bruna Lohmann-Menezes, Jeanine Giarolla, Yasmin da Silva-Santos, Giuliana Petri, Sabrina Epiphanio

**Affiliations:** ^1^Department of Clinical and Toxicological Analysis, School of Pharmaceutical Sciences, University of São Paulo, São Paulo, Brazil; ^2^Department of Pharmacy, School of Pharmaceutical Sciences, University of São Paulo, São Paulo, Brazil; ^3^Animal Facility, FMABC University Center, São Paulo, Brazil

**Keywords:** diclofenac, dog, adverse reactions, nonsteroidal anti-inflammatory drug, toxicity

## Abstract

In many homes worldwide, dogs are considered part of the family. Every possible care is given to animals, including drug treatments. However, many animal guardians, in an attempt to minimize pain or improve the quality of life of their dogs, provide drugs without a veterinarian’s prescription. Diclofenac, a non-steroidal anti-inflammatory drug that acts on cyclooxygenase-1 and cyclooxygenase-2 enzymes, is associated with several adverse events, especially related to the gastrointestinal tract, both in humans and pet animals. Therefore, the availability of information about the effects of this drug in different species is always essential. This narrative review aims to present adverse reactions the domestic dog (*Canis lupus familiaris*) can suffer when exposed to diclofenac. Scientific publications, books, and case reports were consulted, and inquiries were also carried out with regulatory agencies. Many reports of suspected adverse reactions, especially related to the gastrointestinal tract, were found. Other clinical manifestations and lesions were also identified in the cardiovascular system, liver, kidneys, and hematological examinations. Therefore, diclofenac may constitute a hazard to dogs, mainly due to possible damage to the gastrointestinal tract. This fact reinforces the need to seek veterinary advice before providing any drug to animals, in addition to recommendations on ensuring the correct storage of medications to avoid accidental exposure.

## 1 Introduction

The dog was the first domesticated animal. The origin of the species and the close relationship with humans may have begun with the migration of wolves, orientated by the presence of carcasses left by humans. Possibly, this process was followed by coordinated activities, such as hunting and defense. Moreover, agriculture caused divergences between wolf ancestors and dogs since the lifestyle changed and humans started to live sedentarily (1–4).

Dogs are companions in families worldwide and are considered family members, or play a support role assisting patients with disabilities and other medical conditions (5). Additionally, dogs are essential to human well-being and psychosocial health (6), besides dogs working in a variety of functions, such as mobility and therapy assistance, protection, and detection (of narcotics and explosives for example) (7).

**Figure 1:**
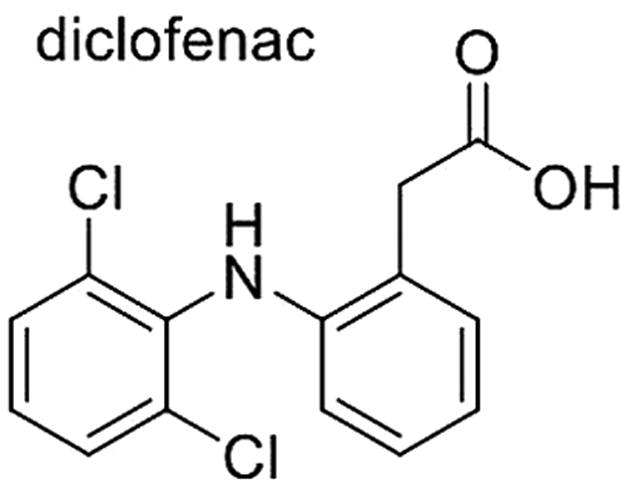
Chemical structure of diclofenac.

Maintaining a dog’s health requires access to veterinary care, but financial barriers, especially post-pandemic, highlight the need for reliable information (8, 9). However, many pet owners rely on the internet for veterinary advice, which may be inaccurate (10–12). Some drugs, like non-steroidal anti-inflammatory drugs (NSAIDs), are safe for humans but potentially harmful to animals (13, 14). Diclofenac (Figure 1), an NSAID commonly used for inflammation, inhibits cyclooxygenase, consequently reducing the production of prostaglandins (15, 16). Therapeutic effects are related to cyclooxygenase-2 inhibition, and gastrointestinal adverse reactions are related to cyclooxygenase-1 inhibition (15, 17). Despite this, the drug it is used in the veterinary field, mainly for swine and cattle, being contraindicated in dogs, as it may result in gastrointestinal and renal toxicity. Studies show diclofenac has a more prolonged circulation in dogs due to enterohepatic recirculation, increasing its toxicity (21, 23). Other species can also suffer adverse effects such as the vultures, which developed renal failure when exposed to the drug through consuming tissues of dead cattle previously treated with the drug (20). Unlike humans, dogs and cats are highly susceptible to NSAIDs, especially due to the high gastrointestinal absorption rate, half-life and extensive enterohepatic cycle (21, 22).

Studies about the diclofenac metabolism in animals determined that, in dogs, 35 to 40% of the substance is excreted in the urine. Another way of drug excretion is through the bile: an ester derivative was found, and it was assumed that its hydrolysis occurs in the duodenum since this derivative was decomposed even in weakly alkaline solutions. This release in the duodenum leads to enterohepatic recirculation, so the drug remains in circulation in the animal for longer (23).

This review stresses the importance of providing accurate, evidence-based information on the risks of diclofenac for pets and advocates for responsible drug use, once the news is spreading on the internet. In addition, we presented regulatory veterinary agencies’ information, reinforcing the importance of rational drug administration.

## 2 Materials and methods

Besides to the literature review, official government websites and health regulatory agencies from different countries were consulted, including Brazil, the United States (Food and Drug Administration, FDA), Chile, Argentina, Peru, France, Portugal, Spain, Italy, and the European Medicines Agency. The search for data from these regulatory agencies was conducted through websites and emails to verify information on the commercialization of diclofenac for veterinary use and records of adverse reactions in dogs during exposure to diclofenac. This review included articles that discussed the exposure of the canine population to diclofenac. The studies on pharmacokinetics, exposure of dogs to combined treatments, and articles that did not report adverse events were excluded from our review.

## 3 Results and discussion

### 3.1 Veterinary pharmacovigilance

Veterinary pharmacovigilance is responsible for evaluating and improving the safety of medicines, for instance, identifying, preventing, and understanding adverse reactions and assessing the potential harmful effects, not only on animals, but also on exposed human beings and the environment. Other topics, such as identification of off-label use, accidental exposure, lack of effectiveness, monitoring of residues (antibiotic residues in milk, for example), and environmental matters are other issues related to the post-marketing surveillance (24).

### 3.2 Data about diclofenac use in different countries and adverse events

Regulatory agencies from different countries were consulted regarding the commercialization of veterinary medications containing diclofenac and questioned about the adverse reactions in dogs. In response, the European Medicines Agency informed that, at that moment, only requests from European citizens or residents of the continent could be attended to, and requested data still needed to be answered.

On the other hand, The Veterinary Medicines Directorate (United Kingdom Regulatory Agency) informed the authors that there is no veterinary medicine containing diclofenac registered in the United Kingdom, and the department responsible for human medicines is not responsible for adverse events in animals. Therefore, they received only one report about a cat which received a human medication containing diclofenac.

Regarding Spain, the AEMPS (*Agencia Española de Medicamentos y Productos Sanitarios*) notified that during the last 5 years, only one notification about diclofenac adverse events was received, which occurred with two cows. Additionally, there is a system called Cimavet, in which it is possible to verify all the veterinary medicines registered in Spain. However, no products containing diclofenac were found for dogs. There were only two veterinary drugs containing diclofenac, developed for swine, bovine and equine use (25). Beyond the Cimavet, the agency provides quarterly and yearly reports containing security alerts; no information about diclofenac was found in those documents.

In Brazil, according to *Ministério da Agricultura, Pecuária e Abastecimento*, a governmental agency, information about adverse events and product registration needs to be obtained directly from the registration holders. Therefore, research was performed on the agency and the SINDAN (*Sindicato Nacional da Indústria de Produtos para a Saúde Animal*) (union that gather companies responsible for around 90% of the veterinary medicine market in Brazil) websites, to verify the registered products containing diclofenac (26). The drugs found mainly were intended for treating cattle, pigs, horses, goats, and sheep. However, three products containing diclofenac were intended for dogs, containing diclofenac in the antibiotic formulation’s diluent. Nonetheless, in the respective leaflets, the concentration of the active in the diluent is not informed. Therefore, the company’s website and contacts were consulted to obtain additional information, though without success.

The other countries did not provide an answer to the question mentioned before. The North American regulatory agency, the Food and Drug Administration (FDA), has a department specialized in veterinary drugs, the Center for Veterinary Medicine, which provides a document called Green Book (27, 28), in which it is possible to verify the approved drugs in the country for veterinary use. Containing the active ingredient diclofenac, only the product Surpass® Topical Anti-Inflammatory Cream was found, which is used topically to control pain and inflammation related to osteoarthritis, being exclusively developed for use in horses (29–31). The FDA also has a veterinary adverse events database (The Animal & Veterinary API Endpoints), which contains information reported from January 1987. The data is available as part of openFDA, a project that enables access to information by the public (30). The database comprises cases reported by veterinarians or pet guardians. The system allows searches through the name of the active ingredient, animal affected, and date of report, among others. A survey was conducted in October 2024 regarding reports containing the word “diclofenac” (30). It is important to emphasize that the situations are adverse reactions, and there is no confirmation of the relationship between diclofenac and the symptoms.

184 occurrences of the word diclofenac in the were found openFDA database. In the documents, not only the suspected drug can be informed, but also the concomitant medication. The search did not consider commercial names of drugs, as the active ingredient diclofenac was used for the investigation. Considering just the cases in which canine species were involved and diclofenac was the suspected drug and not the concomitant, seven cases remained, and three were about accidental exposure (one with the outcome of death). This event reinforces the importance of correctly storing of medicines, which must be kept out of reach of animals and children.

In all reported cases the off-label use field was selected as “species off-label” because, as previously mentioned, the only veterinary drug containing diclofenac registered by the FDA is for equine use. For this review, the suspected adverse events were divided according to the systems and organs affected: gastrointestinal tract, liver, nervous and respiratory systems (Table 1). The death report was not considered, since the causes were not informed. Likewise, accidental exposures were not contemplated, as they are adverse events, but they are not clinical manifestations that occurred during drug exposure. Regarding depression, more details were not provided, being classified as a condition related to the nervous system. The anorexia was included in the gastrointestinal tract, as it was related to other gastrointestinal signs.

**Table 1:** Suspected adverse reactions in dogs.

Affected systems organs	Gastrointestinal tract	Liver	Nervous system	Respiratory system
Suspected adverse reactions	Diarrhea (2) Vomit (3) Anorexia (1) Hypersalivation (1) Change in stool consistency (1) Melena (1)	Hepatitis (2) Liver fat (1) Elevated liver enzymes (1)	Depression (1)	Panting (1)

The table displays the quantities of suspected adverse reactions experienced by dogs, as recorded in the OpenFDA (30) database, from 1987 to October 2024. The data is categorized by organ systems/organs, with the number of reported cases indicated in parentheses.

The gastrointestinal tract was the most affected organ system in dogs with suspected adverse reactions (60% of the reactions). The liver is the second organ affected (26.7%), followed by the respiratory and nervous systems (6.7%). Some biological toxic responses will be briefly discussed in the following paragraphs. Articles retrieved in research databases also presented undesirable outcomes in dogs, possibly related to the use of diclofenac. A case report published in 2017 informs about a dog that received diclofenac 50 mg (route of administration not reported) every 12 h for 13 days. Clinical manifestations associated with the gastrointestinal tract were observed, and the dog developed pure red cell aplasia. The animal presented hematochezia, anorexia, and prostration, which may indicate hemorrhagic gastroenteritis. The clinical manifestations were treated, but the blood count showed persistent non-regenerative anemia (32).

Another report refers to a dog that received 50 mg of diclofenac every 8 h for 2 days. The dog was taken to the veterinary hospital due to extreme sialorrhea and hematochezia. The next day, the animal presented bloody diarrhea, dysphagia, and oliguria, without improvement. The blood analysis revealed mild dehydration and leukocytosis with neutrophilia and monocytosis. On the third day, the animal showed hyporexia, gastric hyperalgesia, and 1 kg weight loss. After outpatient treatment, the animal recovered (33).

A 1996 study conducted experiments in dogs intending to analyze the role of misoprostol and selective vagotomy in protecting the gastrointestinal mucosa. Diclofenac was used as an inducer of injury. The animals were divided into 3 groups with 10 dogs each (one group treated with misoprostol, one with selective vagotomy denervation of the area responsible for the acid secretion of the stomach, and another with both). The groups received diclofenac intramuscularly at a dose of 1 mg/kg. Among all diclofenac-treated dogs, 53.3% (16/30) had stomach lesions and, 33.3% (10/30) showed duodenal lesions. In addition, 70% (21/30) of animals tested positive for fecal occult blood, 30% (9/30) presented melena, and 30% (9/30) had diarrhea. The lesions mainly affected the gastric antrum, and the duodenum was less frequently affected. It is also necessary to consider that these dogs received gastric protection through misoprostol (prostaglandin E1 analog, used as ulcer prevention) and/or vagotomy (which provides gastric acidity reduction) (34). Notwithstanding, diclofenac caused gastrointestinal lesions, despite gastric protective factors and intramuscular administration. Probable mechanisms involved are impaired prostaglandin synthesis, reduced blood flow and, consequently, reduced secretion of bicarbonate and mucus, decreased hydrophobicity of the epithelial layer, impaired cell regeneration, and increased neutrophils adhesion (21).

Freitas et al. (35) reported that a Chow Chow breed dog received three tablets of 50 mg of diclofenac potassium during 48 h due to pain without a medical prescription. After 3 days, despite the improvement in the initial pain, he showed apathy, inappetence, increased abdominal volume, local pain, and pasty and dark stool. The blood analysis revealed leukocytosis, toxic neutrophils, thrombocytosis, and platelet aggregation. Although creatinine and alkaline phosphatase were increased, these changes were not considered possible liver or kidney damage diagnoses due to the lack of any other information indicative of lesions in these organs (35). On the fourth day, he died, and during the necropsy was observed pallor and distension of the abdominal cavity, as well as fibrin deposition and serous and bloody exudate. In addition, a perforated ulcer was identified at the gastroduodenal transition, leading to peritonitis, sepsis and, subsequently, the animal’s death (35).

Also related to canine gastrointestinal mucosal damage, a study analyzed the effects of some NSAIDs in dogs. Those that received diclofenac were orally exposed to 50 mg/kg/day for 3 weeks. Initially, gastroscopy was performed to evaluate the mucosal condition before exposure to the bioactive component and after, repeated once a week for 3 weeks, and samples from the cardiac to pyloric regions were collected for biopsy. The histopathological analyses showed gastric mucosal degeneration, increasing over the days (36). These findings related to gastrointestinal mucosal degeneration corroborated a later study in which eight dogs received 3 mg/kg twice a day of diclofenac orally for 4 days. In addition, at the end of the treatment, the animals had irregular deep ulcers with mucosal necrosis (37).

Prostaglandins in the gastrointestinal tract lead to acid secretion inhibition and increased blood flow, contributing to mucus production and exerting a protective action. Thus, it is known that one of the mechanisms of damage to the gastrointestinal mucosa induced by NSAIDs is due to this reduction in blood flow (21). Furthermore, three studies were conducted in Japan that analyzed gastric blood flow reduction in Beagle dogs induced by diclofenac, administered by suppository. The 1 mg/kg and 12.5 mg doses suppositories reduced blood flow in the gastric body (38, 39, 49).

Besides the stomach, lesions were also observed in the small intestine of dogs exposed to this medication. In a study, dogs were divided into groups, containing five animals: one of the groups received diclofenac 1 mg/kg a day for 10 days, observing mucosal lesions in the entire group. In another group, a segment of the ileum was surgically isolated (with the maintenance of blood flow), and three of five animals showed small and restricted lesions on the intestinal mucosa but not in the isolated part of the ileum. There was also one experimental group (sham group), which underwent only the surgical isolation procedure on the ileum, without drug administration, to demonstrate that the intervention itself did not cause damage to the mucosa (40).

According to Baltoyiannis and collaborators, a possible explanation for the lower number of lesions in the group with an isolated segment of the ileum is the smaller total surface area for absorption in the intestine, reducing the region in which diclofenac is reabsorbed. Isolated portions of the ileum continued to receive blood flow but not enteric content. Thus, it is presumed that intraluminal factors are necessary for the pathogenesis of intestinal lesions, as isolating the ileum from food, bile, pancreatic secretions, and drug metabolites prevents damage (40).

The metabolites of diclofenac are easily hydrolyzable, allowing them to revert to the active form of the drug. Consequently, in the presence of enteric content, they can return to their active form and be reabsorbed (23). A study using 14C-labelled diclofenac to dogs suggested that the main metabolite (diclofenac acyl-glucuronide, 80% of total radioactivity in the bile) is hydrolyzed in the intestine and participates enterohepatic circulation. Diclofenac acyl-glucuronide was hydrolyzed by weak alkaline and beta-glucuronidase, and when administered intraduodenally, it was absorbed and subsequently excreted in the bile once more (41). NSAIDs increase the permeability of the intestinal mucosa, exacerbating the effects of enzymes, bile salts, bacteria and enteric content associated, which can cause severe injuries. It is clear that regardless of the administration route, the gastrointestinal tract can be affected. The results obtained in this experiment can be correlated with previous observations regarding the importance of enterohepatic circulation in the pathogenesis of diclofenac toxicity in dogs. It was possible to observe that diclofenac and its metabolites in the intestine are necessary for the occurrence of lesions in the region (40). On the other hand, the use of diclofenac in ophthalmic solutions resulted in elevated blood concentrations of the drug, leading to gastrointestinal erosions (19) and hemorrhages in 5% of the treated dogs (42).

Renal alterations were also observed. The exposure of eight dogs to diclofenac (oral) 3 mg/kg twice a day for 4 days increased creatinine and urea levels in the blood. In addition, the histopathology exhibits renal tubule distention, indicating mild nephropathy (37). In another investigation, the drug caused canine kidney death cells, starting with apoptosis pathways, and then the process changed to necrosis after a long period of exposure to the drug (43).

The exposure of dogs to diclofenac was linked to liver abnormalities. Selvaraj et al. conducted studies on immunoallergic hepatitis in dogs (44, 45). Beagle dogs treated with high doses of diclofenac (1 or 3 mg/kg/day for 28 days) showed liver function test abnormalities and histopathology revealed hepatic steatosis, glycogen depletion, hepatocyte apoptosis, acute lobular hepatitis, granulomas, and mastocytosis. Genome scans indicated stress, immune response, and inflammation. Hematological changes included reduced hematocrit and hemogloetbin but increased reticulocytes, white blood cells, platelets, neutrophils, and eosinophils. Myeloperoxidase induction and oxidative stress were also noted (45). The study concluded that failures in canine immune response programming lead to allergic hepatitis and liver granulomas (44).

The cardiovascular system was also studied after diclofenac exposure. A study using a preparation of canine right ventricular muscle cells and Purkinje fibers found that high-dose diclofenac combined with a potassium channel blocker increased the risk of arrhythmia. In normal hearts, even high doses did not influence repolarization and arrhythmia risk. The concentrations used in the experiment were slightly higher than the therapeutic blood level, approximately 2–7 mM/L, based on data after oral administration of 50 mg diclofenac (46).

Another study aimed to verify the relationship between NSAIDs use and worsening angina episodes. Dogs were exposed to 1 mg/kg, 3 mg/kg, and 10 mg/kg of diclofenac twice daily for 4 days. In addition, they also received nitroglycerin as a vasodilator. All treated groups developed venoconstriction, of marginal significance. Although this venoconstriction was of minor significance, a point of attention was raised. Considering a large population’s long-term use of diclofenac, this change may represent risks in a real exposure situation (47).

## 4 Conclusion

Data from the openFDA database and literature indicate that administering diclofenac to dogs can damage various organ systems, with the gastrointestinal tract being particularly vulnerable. Diclofenac exposure in dogs can be accidental or intentional, and the reported cases of adverse reactions may represent only “the tip of the iceberg” since many veterinary professionals do not report incidents, especially when the drug is used off-label or administered by owners without prescription. Veterinary evaluation is crucial for selecting appropriate treatments and raising awareness about the risks of medicating animals without guidance, particularly in a digital age where misinformation is widespread.
